# Supplementary material for: Spatial protein analysis in developing tissues: a sampling-based image processing approach
Source: Philos Trans R Soc Lond B Biol Sci. 2020 Aug 24;375(1809):20190560. doi: 10.1098/rstb.2019.0560 (PMC7482225; doi:10.1098/rstb.2019.0560)
Supplement: YAP and CDX2 distribution [file rstb20190560supp1.pdf]

PHILOSOPHICAL TRANSACTIONS B

Phil. Trans. R. Soc. B.

Spatial protein analysis in developing tissues: a sampling-based image processing approach

Karolis Leonavicius, Christophe Royer, Antonio M. A. Miranda, Richard Tyser, Annemarie Kip and Shankar Srinivas*

Department of Physiology Anatomy and Genetics, University of Oxford, Oxford OX1 3QX, UK

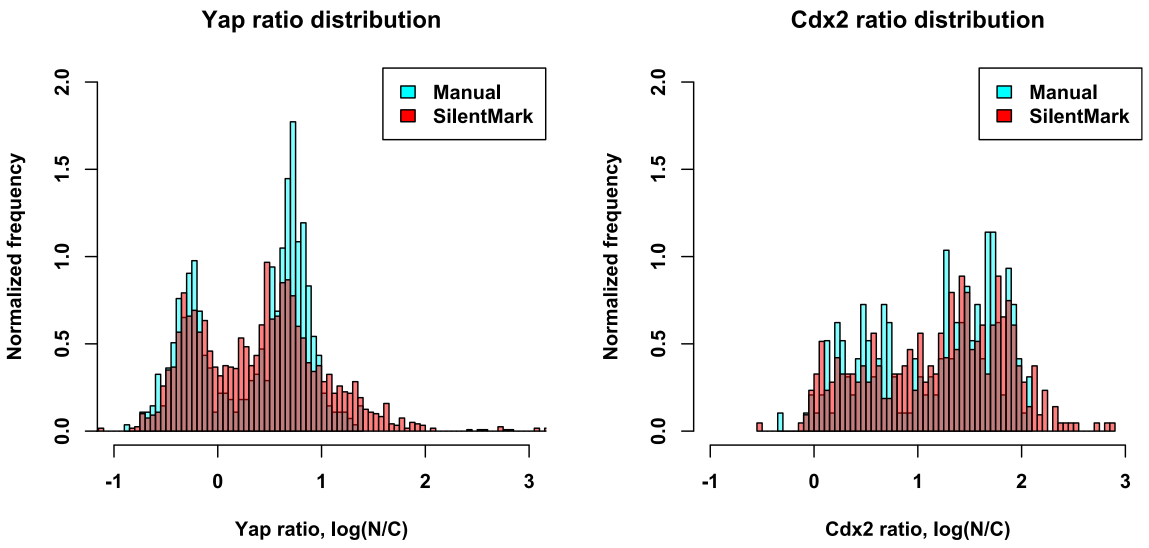

Supplementary Figure S1: Comparison of manual cell outlining and SilentMark performance. Distributions of measured values of Yap and Cdx2 nuclear/cytoplasmic ratios.
